# PAPP‐A: a promising therapeutic target for healthy longevity

**DOI:** 10.1111/acel.12564

**Published:** 2016-12-29

**Authors:** Cheryl A. Conover, Claus Oxvig

**Affiliations:** ^1^ EndocrinologyMayo ClinicRochesterMNUSA; ^2^ Department of Molecular Biology and GeneticsAarhus UniversitetAarhusDenmark

**Keywords:** aging, IGF, PAPP‐A

## Abstract

Pregnancy‐associated plasma protein‐A (PAPP‐A) is a proteolytic enzyme that was discovered to increase local insulin‐like growth factor (IGF) availability for receptor activation through cleavage of inhibitory IGF binding proteins (IGFBPs). Reduced IGF signaling has been associated with increased lifespan and healthspan. Therefore, inhibition of PAPP‐A represents a novel approach to indirectly decrease the availability of bioactive IGF. Here, we will review data in support of PAPP‐A as a therapeutic target to promote healthy longevity.

## Introduction

PAPP‐A was first identified as a placental protein of primates, but no biochemical function was known prior to our discovery in 1999 that PAPP‐A is a proteolytic enzyme. We showed that cultured human fibroblasts secrete PAPP‐A, and that PAPP‐A cleaves IGFBP‐4 (Lawrence *et al*., 1999). Importantly, IGFBP‐4 becomes a PAPP‐A substrate only following its binding of IGF‐I or ‐II. The two proteolytic fragments resulting from PAPP‐A cleavage have very low affinity for the IGFs, causing the IGFBP‐4/IGF complex to dissociate.

Further studies found PAPP‐A to be expressed in many different cells types besides fibroblasts, including human osteoblasts, vascular smooth muscle cells, pre‐adipocytes, ovarian granulosa cells, and kidney mesangial cells (Lawrence *et al*., 1999; Conover *et al*., 2001, 2004b, 2006; Resch *et al*., 2004). It was characterized as a metalloproteinase of the metzincin superfamily and defined a new subfamily, the pappalysins, distinct from previously recognized subfamilies including the matrix metalloproteinases (Boldt *et al*., 2001). Since the discovery of its function, PAPP‐A and its proteolytic activity have been studied in a diverse range of species, including human, baboon, cow, pig, mouse, naked mole rat, marsupial, and zebrafish (Mazerbourg *et al*., 2001; Conover *et al*., 2004a; Tchoukalova *et al*., 2009; Juengel *et al*., 2010; Phang *et al*., 2010; Kjaer‐Sorensen *et al*., 2013; Brohus *et al*., 2015). Based on genomic data, a gene encoding PAPP‐A appears to be present in all vertebrates.

**Figure 1:**
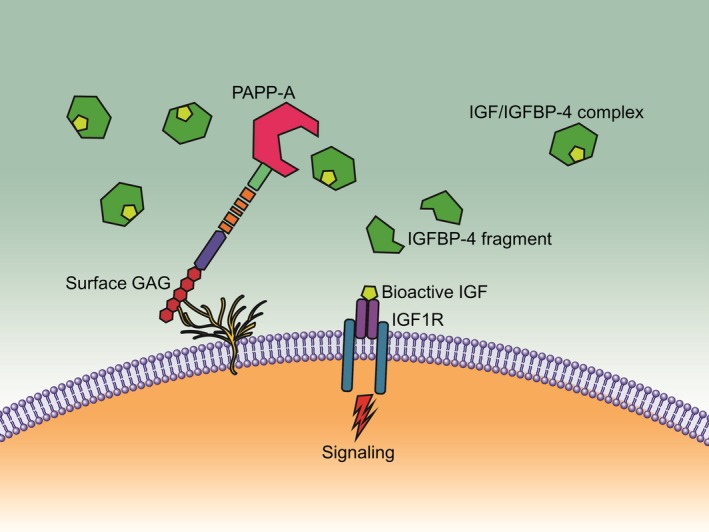
Role of PAPP‐A and IGFBP‐4 in the local control of IGF signaling.

The main known function of PAPP‐A is to increase local IGF bioavailability through cleavage of inhibitory IGFBPs, in particular IGFBP‐4. Indeed, PAPP‐A is probably the only physiological IGFBP‐4 proteinase (Conover *et al*., 2004a; Laursen *et al*., 2007; Ning *et al*., 2008; Conover, 2012; Oxvig, 2015). Secreted PAPP‐A tethers to cells through binding to surface glycosaminoglycans (Fig. 1). IGF bound to IGFBP‐4 is not bioactive. However, the liberation of bioactive IGF upon cleavage of IGFBP‐4 in the pericellular environment initiates IGF signaling. PAPP‐A‐induced enhancement of local IGF action through proteolysis of IGFBP‐4 has been demonstrated *in vitro* and *in vivo* in several different systems (Conover, 2012; Oxvig, 2015). The only exception to date is in early fetal development of zebrafish (Kjaer‐Sorensen *et al*., 2013). Curiously, a paralog of IGFBP‐4 appears to be lacking in zebrafish (Li *et al*., 2009).

Reduced IGF signaling has been associated with longevity and increased healthspan (Katic & Kahn, 2005). Therefore, a reduction in PAPP‐A proteolytic activity represents a novel approach to indirectly decrease the availability of bioactive IGF. For therapeutic intervention, such a strategy is expected to moderately restrain IGF signaling and hence cause fewer adverse effects compared to direct inhibition by targeting the IGF receptor. Here, data in support for the development of PAPP‐A as a therapeutic target are reviewed. These are principally based on studies with genetically modified mice and a recently developed monoclonal antibody specific for PAPP‐A, which selectively inhibits PAPP‐A cleavage of IGFBP‐4 (Mikkelsen *et al*., 2008, 2014). The focus of this review is on curbing aging and age‐related diseases.

## PAPP‐A and lifespan studies

Both male and female PAPP‐A knockout (KO) mice on chow diet live 30–40% longer than wild‐type (WT) littermates, with no secondary endocrine abnormalities (Conover *et al*., 2010b). Circulating levels of growth hormone (GH), IGF‐I, glucose, and insulin were not significantly different between PAPP‐A KO and WT mice in this study. PAPP‐A KO mice also live longer when fed a high fat diet starting as adults (Conover *et al*., 2015). Thus, PAPP‐A deficiency can promote longevity without dietary restriction. Furthermore, this extended lifespan is not a secondary consequence of a small body size because PAPP‐A KO mice rescued from the dwarf phenotype by enhanced IGF‐II expression during fetal development retain their longevity advantage (Conover *et al*., 2010a). Finally, conditional knockout of the PAPP‐A gene in adult mice using tamoxifen‐inducible Cre recombinase methodology (Conover *et al*., 2013a) also resulted in a 20% extension of lifespan (*P *<* *0.0001; manuscript in preparation). End‐of‐life pathology showed delayed occurrence of fatal neoplasias and indicated decreased incidence and severity of conditions with age‐related degenerative changes, such as cardiomyopathy, nephropathy, and thymic atrophy in PAPP‐A KO mice compared to WT littermates (Conover *et al*., 2010b).

Several mouse models with reduced GH‐stimulated IGF‐I expression by liver and low levels of circulating IGF‐I (Snell, Ames dwarf, GH receptor KO) have also been found to have extended longevity (Junnila *et al*., 2013). On the other hand, transgenic mice over‐expressing GH exhibit a shortened lifespan (Bartke, 2003). It is important to note that PAPP‐A KO mice have normal levels of circulating IGF‐I (and GH) and their phenotype reflects reduction in local IGF action. Unlike the GH mutant mice that have postnatal growth retardation, deletion of the PAPP‐A gene manifests itself early in fetal development as proportional dwarfism (Conover *et al*., 2004a). The lifespan extension in the Snell, Ames dwarf, and GH receptor KO models reflects GH tone rather than IGF‐I bioavailability.

## PAPP‐A and age‐related diseases

### Thymic involution

Thymic involution, the shrinking and morphological changes of the thymus in aging vertebrates, results in immune senescence that has the potential to impact aging and age‐related diseases. As reported previously, the thymi of PAPP‐A KO mice are relatively resistant to normal age‐dependent involution (Vallejo *et al*., 2009). At 18 months of age, thymi of WT mice are markedly reduced in size and cellularity with extensive adipose infiltration. In contrast, the similarly aged PAPP‐A KO mice maintain the lobular thymic structure and cellularity and the ability to produce diverse immune‐competent T cells.

We recently completed a study of young PAPP‐A KO and WT mice that were fed a high fat diet for 20 weeks. Compared to WT littermates, the thymi of male and female PAPP‐A KO mice were 122% (*P *=* *0.007) and 158% (*P *<* *0.0001) larger, respectively. Even with the larger size, the thymi of female PAPP‐A KO mice contained 40% less lipid (*P *=* *0.033) than WT mice. In a preliminary study, once a week intraperitoneal treatment of WT mice on high fat diet with an inhibitory monoclonal antibody against PAPP‐A (mAb‐PA) resulted in thymic size that was 120% of treatment with isotype control antibody. Targeting PAPP‐A to reduce/delay thymic involution could, therefore, play a central role in regulating immune competence and healthy aging. However, further studies are needed to better characterize and understand the role of PAPP‐A in thymic biology.

### Atherosclerosis

As first reported by Bayes‐Genis *et al*. (2001), PAPP‐A expression is increased in unstable human atherosclerotic plaques associated with activated macrophages, smooth muscle cells, and endothelium. To assess whether this increased PAPP‐A expression was cause or consequence of lesion development, we studied apolipoprotein E (ApoE) KO mice, which are an established murine model of atherosclerosis (Breslow, 1996). Reduction in local IGF bioavailability through PAPP‐A gene deletion in ApoE KO mice inhibited atherosclerotic plaque burden by 70–80% following 20 weeks on a high fat diet. There was no effect on macrophage recruitment or expression of pro‐inflammatory cytokines, and no secondary endocrine effects were observed (Harrington *et al*., 2007). Tang *et al*. (2012) suggested that PAPP‐A inhibits cholesterol efflux in macrophage‐derived foam cells through the IGF‐I signaling pathway, thus contributing to the pathogenesis of atherosclerosis.

To assess a possible effect of PAPP‐A gene knockout during development, a similar experiment was carried out in ApoE KO mice with the PAPP‐A gene ‘floxed’ and crossed with mice with tamoxifen‐inducible Cre recombinase (Conover *et al*., 2013a). Inducible reduction in PAPP‐A gene expression in adult ApoE KO mice significantly inhibited established atherosclerotic plaque progression and the development of advanced plaque with necrotic cores (Bale *et al*., 2014). In this inducible model, reduced PAPP‐A expression was not limited to the vasculature, and changes in other tissues could potentially contribute to the observed effects. PAPP‐A gene expression was efficiently reduced in thymus and spleen (Conover *et al*., 2013a), which could dampen the negative effects of the immune response in atherosclerosis (Hansson & Libby, 2006). Moreover, weekly treatment (intraperitoneal injections) of ApoE KO mice with mAb‐PA, which specifically inhibits PAPP‐A‐mediated IGFBP‐4 proteolysis, similarly inhibited atherosclerotic plaque progression (Conover *et al*., 2016b). This latter study was the first to demonstrate proof of principle and provide translational support for the development of novel therapeutic strategies to inhibit human atherosclerosis by targeting the proteolytic activity of PAPP‐A.

It is of note that PAPP‐A can also cleave IGFBP‐5 (Laursen *et al*., 2007). However, as there are other proteases that have IGFBP‐5 as substrate, the impact of PAPP‐A‐specific IGFBP‐5 proteolysis is difficult to ascertain *in vivo*. The inhibitory mAb‐PA was selected to target a specific region of PAPP‐A required for proteolytic cleavage of IGFBP‐4, but not IGFBP‐5 (Boldt *et al*., 2004; Mikkelsen *et al*., 2008, 2014), a so‐called substrate‐binding exosite. Therefore, mAb‐PA inhibits proteolysis of IGFBP‐4, but not IGFBP‐5. This immunoneutralizing antibody was effective in inhibiting atherosclerotic plaque progression (Conover *et al*., 2016b). In addition, ApoE KO mice expressing a mutated PAPP‐A, which selectively lacks proteolytic activity against IGFBP‐4 and not IGFBP‐5, did not show accelerated atherosclerotic lesion development compared to mice overexpressing native PAPP‐A (Boldt *et al*., 2013). Interestingly, neither did ApoE KO mice expressing proteolytically active PAPP‐A, but lacking domains for cell association. Thus, data so far support the model set forth by Laursen *et al*. (2007) that although cleavage of both IGFBP‐4 and IGFBP‐5 is required for release of bioactive IGF, cell surface‐localized proteolysis of IGFBP‐4 represents the final regulatory step of efficient IGF delivery to receptors.

### Visceral obesity

In contrast to subcutaneous fat, the accumulation of visceral fat is strongly associated with severe metabolic complications in humans (Wajchenberg, 2000; Jensen, 2008; Perrini *et al*., 2008), which shows differential expression of PAPP‐A in these two types of adipose tissue (Tchoukalova *et al*., 2009; Davidge‐Pitts *et al*., 2014). Mouse adipose tissue shows a similar difference with fivefold to 10‐fold higher expression of PAPP‐A mRNA in visceral (mesenteric) fat compared to subcutaneous (inguinal, subscapular) fat. There was a preferential impact of PAPP‐A deficiency in mice on a high fat diet to prevent increases in mesenteric adipocyte size with no significant effect on subcutaneous fat depots, probably due to the high expression of PAPP‐A in mesenteric fat (Conover *et al*., 2013b). The decrease in accumulation of mesenteric adipose tissue was associated with increased expression of adiponectin, which has anti‐inflammatory and cardio‐protective effects and enhanced insulin receptor signaling in this depot (Conover *et al*., 2013b). For these reasons, PAPP‐A may be a potential target for treatment and/or prevention strategies for visceral obesity and related morbidities.

### Fragility

Aging is associated with loss of skeletal muscle mass and, consequently, compromised muscle function (Marzetti *et al*., 2009). It has been found that PAPP‐A KO mice are resistant to skeletal muscle aging (Conover *et al*., 2016a). In this study, top genes regulated in muscle from 18‐month‐old PAPP‐A KO compared to WT mice were associated with increased muscle function, increased lipid metabolism, and decreased stress. Fiber cross‐sectional area and intrinsic mitochondrial oxidative capacity were significantly increased in skeletal muscle of aged PAPP‐A KO compared to WT mice. Moreover, these 18‐month‐old PAPP‐A KO mice exhibited significantly enhanced endurance running on a treadmill. Also, PAPP‐A KO mice are resistant to age‐related metabolic dysfunction (Hill *et al*., 2015), largely driven by skeletal muscle.

These studies may seem contrary to other studies where PAPP‐A overexpression in skeletal muscle increased muscle mass in response to Injury (Rehage *et al*., 2007). It is known that enhanced IGF signaling is important for muscle regeneration in response to injury (Clemmons, 2009). However, this response could be different in aging muscle. One possibility is that reduced IGF signaling in pluripotent stem cells residing in skeletal muscle protects against premature depletion. More work is required to delineate the roles of IGFs in this tissue.

### Nephropathy

The kidney has been suggested to be an organ of particular interest for targeted PAPP‐A inhibition (Swindell *et al*., 2010). At 18 months of age, there was a higher incidence and severity of chronic nephropathy in WT compared to PAPP‐A KO mice (Conover *et al*., 2010b). It was also found that PAPP‐A KO mice are resistant to the development of diabetic nephropathy (Mader *et al*., 2013). In this model of type 2 diabetes, increased glomerular size and thickened Bowman's capsules were observed in WT mice, but not in PAPP‐A KO mice. Glomerular expression of PAPP‐A was increased in kidneys of patients with diabetic nephropathy (Mader *et al*., 2013). Studies in cultured human mesangial cells indicated potent upregulation of PAPP‐A mRNA and protein expression by pro‐inflammatory cytokines (Donegan *et al*., 2016), which are prevalent in diabetic nephropathy.

### Cancer

The involvement of PAPP‐A proteolytic activity in cancer is increasingly suggested. For example, clinical data show that PAPP‐A serum levels are elevated in patients with lung cancer (Bulut *et al*., 2009), and PAPP‐A can promote lung cancer growth *in vivo* (Pan *et al*. 2012). PAPP‐A is highly expressed in aggressive forms of human breast cancer (Mansfield *et al*., 2014). Interestingly, Chander *et al*. (2011) found that mutation of the tumor suppressor gene, p53, increases PAPP‐A transcription in breast cancer cell lines and mammary gland tissues.

In agreement with such findings, several publications indicate that targeting of PAPP‐A proteolytic activity is relevant to the prevention of cancer growth and metastases. Overexpression of proteolytically active, but not proteolytically inactive, PAPP‐A in an ovarian cancer cell line was shown to have increased tumor aggressiveness *in vivo* (Boldt & Conover, 2011). The first report of mAb‐PA efficacy was in lung cancer cell xenografts expressing PAPP‐A (Mikkelsen *et al*., 2014). PAPP‐A is also overexpressed in a subset of human ovarian tumors, and weekly treatment of mice harboring these intraperitoneal patient tumorgrafts with the inhibitory antibody, mAb‐PA, could reduce tumor size and ascites burden. Treatment with mAb‐PA also conferred chemo‐sensitivity to a high PAPP‐A‐expressing chemo‐resistant patient tumor (Becker *et al*., 2015). These beneficial effects of inhibiting the proteolytic activity of PAPP‐A were only manifested in tumors expressing moderate‐to‐high levels of PAPP‐A. Thus, PAPP‐A expression in patient tumors, or ascites (Thomsen *et al*., 2015), may be a biomarker for patient response to PAPP‐A therapies. We also have unpublished data indicating that mAb‐PA treatment can prevent ovarian tumor metastases to intraperitoneal lining and perigonadal fat, these being prime sites for metastases via ascites. This fits with the identification of PAPP‐A as a migration/invasion‐promoting gene in an ovarian cancer cell line (Boldt & Conover, 2011), malignant pleural mesothelioma cells (Huang *et al*. 2013), nonsmall cell lung cancer (Salim *et al*., 2013), and melanoma (Prithviraj *et al*. 2015).

## Inflammation

Aging and many age‐related diseases are associated with inflammation. However, the use of anti‐inflammatory drugs to alleviate these conditions is problematic because they affect multiple pathways and lack specificity. For example, cyclooxygenase‐2 inhibition was used to counteract inflammation, but was associated with serious cardiovascular complications (Sohn & Krotz, 2006). Furthermore, inflammation is important in normal tissue repair and defense against infection. Thus, it is generally recommended that targets should be specific and downstream of inflammatory pathways (Gauldie, 2007). PAPP‐A could be such a target.

The pro‐inflammatory cytokines, interleukin‐1β (IL‐1β), and tumor necrosis factor‐α (TNF‐α) are potent stimulators of PAPP‐A expression in human fibroblasts, osteoblasts, arterial smooth muscle cells, endothelial cells, pre‐adipocytes, intervertebral disk cells, and mesangial cells (Conover *et al*., 2004b, 2006, 2008; Resch *et al*., 2004; Gruber *et al*., 2013; Davidge‐Pitts *et al*., 2014). In atherosclerotic plaque, macrophage‐derived pro‐inflammatory cytokines act in a paracrine manner to stimulate vascular smooth muscle and endothelial cells to secrete PAPP‐A (Conover *et al*., 2007). This represents an important amplification point in plaque progression, as PAPP‐A can feedback back on activated macrophages and smooth muscle cells to promote IGF‐mediated lipid uptake. With the loss of the PAPP‐A activity induced by pro‐inflammatory cytokines, the vicious cycle would be blunted in spite of the continued presence of macrophages and similar levels of IL‐1β and TNF‐α expression. We suggest that this model of PAPP‐A and inflammatory stress may also apply to other conditions. Aging, associated with chronic low‐grade inflammation, is one such condition. With visceral fat representing a highly active inflammatory microenvironment, especially in obese subjects, adipose tissue dysregulation is another.

## Contraindications for PAPP‐A‐directed therapies

Low circulating PAPP‐A has been associated with adverse effects on placental function and fetal growth in humans (Smith *et al*., 2002). Although the role of PAPP‐A in human pregnancy is not understood, PAPP‐A is believed to be important for placental development. Therefore, targeting PAPP‐A during human pregnancy is not likely to be a viable strategy. Interestingly, placental development in mice does not depend on PAPP‐A (Qin *et al*., 2002; Soe *et al*., 2002).

The involvement of PAPP‐A in normal tissue repair processes also suggests a possible need to suspend PAPP‐A targeting temporarily during such conditions. For example, PAPP‐A increases bone accretion primarily by increasing IGF bioavailability important for prepubertal bone growth (Mohan *et al*., 2003; Qin *et al*., 2006). Fracture repair in PAPP‐A KO mice is temporally compromised, but not prevented from normal resolution (Miller *et al*., 2007). Similarly, controlled increases in PAPP‐A expression are seen in healing human skin (Chen *et al*., 2003), indicating that wound healing may be delayed as a consequence of PAPP‐A targeting.

## Conclusion

Experimental evidence is accumulating that inhibition of PAPP‐A has the potential to promote healthy longevity. It is clearly advantageous that targeting of PAPP‐A has the benefit of a single intervention that affects multiple adverse changes with age, not just a single condition (Figueira *et al*., 2016). PAPP‐A is present in the extracellular environment, and its activity is therefore amenable to pharmacologic intervention. Strategies to inhibit PAPP‐A have recently been developed and tested in experimental models (Mikkelsen *et al*., 2008, 2014; Becker *et al*., 2015; Conover *et al*., 2016b). Rather than the active site of PAPP‐A, a unique substrate‐binding exosite, critical for proteolytic cleavage of IGFBP‐4, is targeted. This efficiently eliminates activity toward IGFBP‐4, but does not interfere with cleavage of other possible substrates of PAPP‐A. Inhibition will target discrete conditions with increased PAPP‐A activity, resulting in moderate restraint of IGF signaling and minimizing side effects. However, much remains to be learned about stages in life at which mice, and possibly humans, are susceptible to improvements in long‐term health by manipulation of PAPP‐A.

## Author contributions

Drs. Conover and Oxvig contributed equally to the preparation of this review.

## Funding

This work was supported by NIA grant AG028141 (CAC) and The Novo Nordisk Foundation (CO).

## Conflict of interest

None declared.

## References

[acel12564-bib-0001] Bale LK , Chakraborty S , Conover CA (2014) Inducible reduction in pregnancy‐associated plasma protein‐A gene expression inhibits established atherosclerotic plaque progression in mice. Endocrinology 155, 1184–1187. 24506074 10.1210/en.2013-2110 PMC3959602

[acel12564-bib-0002] Bartke A (2003) Can growth hormone (GH) accelerate aging? Evidence from GH‐transgenic mice. Neuroendocrinology 78, 210–216. 14583653 10.1159/000073704

[acel12564-bib-0003] Bayes‐Genis A , Conover CA , Overgaard MT , Bailey KR , Christiansen M , Holmes DR Jr , Virmani R , Oxvig C , Schwartz RS (2001) Pregnancy‐associated plasma protein‐A and diagnosis of acute coronary syndromes. N. Engl. J. Med. 345, 1022–1029. 11586954 10.1056/NEJMoa003147

[acel12564-bib-0004] Becker MA , Haluska P Jr , Bale LK , Oxvig C , Conover CA (2015) A novel neutralizing antibody targeting pregnancy‐associated plasma protein‐A inhibits ovarian cancer growth and ascites accumulation in patient mouse tumorgrafts. Mol. Cancer Ther. 14, 973–981. 25695953 10.1158/1535-7163.MCT-14-0880 PMC4394033

[acel12564-bib-0005] Boldt HB , Conover CA (2011) Overexpression of pregnancy‐associated plasma protein‐A in ovarian cancer cells promotes tumor growth *in vivo* . Endocrinology 152, 1470–1478. 21303951 10.1210/en.2010-1095

[acel12564-bib-0006] Boldt HB , Overgaard MT , Laursen LS , Weyer K , Sottrup‐Jensen L , Oxvig C (2001) Mutational analysis of the proteolytic domain of pregnancy‐associated plasma protein‐A (PAPP‐A): classification as a metzincin. Biochem. J. 358, 359–367. 11513734 10.1042/0264-6021:3580359 PMC1222068

[acel12564-bib-0007] Boldt HB , Kjer‐Sorensen K , Overgaard MT , Weyer K , Poulsen CB , Sottrup‐Jensen L , Conover CA , Giudice LC , Oxvig C (2004) The Lin12‐notch repeats of preganancy‐associated plasma protein‐A bind calcium and determine its proteolytic specificity. J. Biol. Chem. 279, 38525–38531. 15262980 10.1074/jbc.M405222200

[acel12564-bib-0008] Boldt HB , Bale LK , Resch ZT , Oxvig C , Overgaard MT , Conover CA (2013) Effects of mutated pregnancy‐associated plasma protein‐a on atherosclerotic lesion development in mice. Endocrinology 154, 246–252. 23161866 10.1210/en.2012-1552 PMC3529381

[acel12564-bib-0009] Breslow JL (1996) Mouse models of atherosclerosis. Science 272, 685–688. 8614828 10.1126/science.272.5262.685

[acel12564-bib-0010] Brohus M , Gorbunova V , Faulkes CG , Overgaard MT , Conover CA (2015) The insulin‐like growth factor system in the long‐lived naked mole‐rat. PLoS ONE 10, e0145587. 26694858 10.1371/journal.pone.0145587 PMC4694111

[acel12564-bib-0011] Bulut I , Coskun A , Ciftci A , Cetinkaya E , Altiay G , Caglar T , Gulcan E (2009) Relationship between pregnancy‐associated plasma protein‐A and lung cancer. Am. J. Med. Sci. 337, 241–244. 19365167 10.1097/MAJ.0b013e31818967a3

[acel12564-bib-0012] Chander H , Halpern M , Resnick‐Silverman L , Manfredi JJ , Germain D (2011) Skp2B overexpression alters a prohibitin‐p53 axis and the transcription of PAPP‐A, the protease of insulin‐like growth factor binding protein 4. PLoS ONE 6, e22456. 21829624 10.1371/journal.pone.0022456 PMC3150379

[acel12564-bib-0013] Chen B‐K , Leiferman KM , Pittelkow MR , Overgaard MT , Oxvig C , Conover CA (2003) Localization and regulation of pregnancy associated plasma protein‐A expression in healing human skin. J. Clin. Endocrinol. Metab. 88, 4465–4471. 12970325 10.1210/jc.2003-030193

[acel12564-bib-0014] Clemmons DR (2009) Role of IGF‐I in skeletal muscle mass maintenance. Trends Endocrinol. Metab. 20, 349–356. 19729319 10.1016/j.tem.2009.04.002

[acel12564-bib-0015] Conover CA (2012) Key questions and answers about pregnancy‐associated plasma protein‐A. Trends Endocrinol. Metab. 23, 242–249. 22463950 10.1016/j.tem.2012.02.008 PMC3348390

[acel12564-bib-0016] Conover CA , Faessen GF , Ilg K , Chandrasekher YA , Christiansen M , Overgaard MT , Oxvig C , Giudice LC (2001) Pregnancy‐associated plasma protein‐A is the insulin‐like growth factor binding protein‐4 protease secreted by human ovarian granulosa cells and is a marker of dominant follicle selection and the corpus luteum. Endocrinology 142, 2155–2158. 11316785 10.1210/endo.142.5.8286

[acel12564-bib-0017] Conover CA , Bale LK , Overgaard MT , Johnstone EW , Laursen UH , Fuchtbauer E‐M , Oxvig C , van Deursen J (2004a) Metalloproteinase pregnancy‐associated plasma protein A is a critical growth regulatory factor during fetal development. Development 131, 1187–1194. 14973274 10.1242/dev.00997

[acel12564-bib-0018] Conover CA , Chen B‐K , Resch ZT (2004b) Regulation of pregnancy‐associated plasma protein‐A expression n cultured human osteoblasts. Bone 34, 297–302. 14962808 10.1016/j.bone.2003.10.011

[acel12564-bib-0019] Conover CA , Bale LK , Harrington SC , Resch ZT , Overgaard MT , Oxvig C (2006) Cytokine stimulation of pregnancy‐associated plasma protein A expression in human coronary artery smooth muscle cells: inhibition by resveratrol. Am. J. Physiol. Cell Physiol. 290, C183–C188. 16338976 10.1152/ajpcell.00199.2005

[acel12564-bib-0020] Conover CA , Harrington SC , Bale LK , Oxvig C (2007) Surface association of pregnancy‐associated plasma protein‐A accounts for its colocalization with activated macrophages. Am. J. Physiol. Heart Circ. Physiol. 292, H994–H1000. 17040968 10.1152/ajpheart.00798.2006

[acel12564-bib-0021] Conover CA , Harrington SC , Bale LK (2008) Differential regulation of pregnancy associated plasma protein‐A in human coronary artery endothelial cells and smooth muscle cells. Growth Horm. IGF Res. 18, 213–220. 17936662 10.1016/j.ghir.2007.09.001 PMC2396756

[acel12564-bib-0022] Conover CA , Bale LK , Grell JA , Mader JR , Mason MA (2010a) Longevity is not influenced by prenatal programming of body size. Aging Cell 9, 647–649. 20550518 10.1111/j.1474-9726.2010.00589.x PMC3072285

[acel12564-bib-0023] Conover CA , Bale LK , Mader JR , Mason MA , Keenan KP , Marler RJ (2010b) Longevity and age‐related pathology of mice deficient in pregnancy‐associated plasma protein‐A. J. Gerontol. A Biol. Sci. Med. Sci. 65, 590–599. 20351075 10.1093/gerona/glq032 PMC2869530

[acel12564-bib-0024] Conover CA , Bale LK , Powell DR (2013a) Inducible knock out of pregnancy‐associated plasma protein‐A gene expression in the adult mouse: effect on vascular injury response. Endocrinology 154, 2734–2738. 23748359 10.1210/en.2013-1320 PMC3713220

[acel12564-bib-0025] Conover CA , Harstad SL , Tchkonia T , Kirkland JL (2013b) Preferential impact of pregnancy‐associated plasma protein‐A deficiency on visceral fat in mice on high‐fat diet. Am. J. Physiol. Endocrinol. Metab. 305, E1145–E1153. 24045868 10.1152/ajpendo.00405.2013 PMC3840208

[acel12564-bib-0026] Conover CA , Bale LK , Marler RJ (2015) Pregnancy‐associated plasma protein‐A deficiency improves survival of mice on a high fat diet. Exp. Gerontol. 70, 131–134. 26325589 10.1016/j.exger.2015.08.007 PMC4600682

[acel12564-bib-0027] Conover CA , Bale LK , Nair KS (2016a) Comparative gene expression and phenotype analyses of skeletal muscle from aged wild‐type and PAPP‐A‐deficient mice. Exp. Gerontol. 80, 36–42. 27086066 10.1016/j.exger.2016.04.005 PMC4893884

[acel12564-bib-0028] Conover CA , Bale LK , Oxvig C (2016b) Targeted inhibition of pregnancy‐associated plasma protein‐A activity reduces atherosclerotic plaque burden in mice. J. Cardiovasc. Transl. Res. 9, 77–79. 26733326 10.1007/s12265-015-9666-9

[acel12564-bib-0029] Davidge‐Pitts C , Escande CJ , Conover CA (2014) Preferential expression of PAPP‐A in human preadipocytes from omental fat. J. Endocrinol. 222, 87–97. 24781252 10.1530/JOE-13-0610 PMC4104415

[acel12564-bib-0030] Donegan D , Bale LK , Conover CA (2016) PAPP‐A in normal human mesangial cells: effect of inflammation and factors related to diabetic nephropathy. J. Endocrinol. 231, 71–80. 27519211 10.1530/JOE-16-0205

[acel12564-bib-0031] Figueira I , Fernandes A , Mladenovic A , Lopez‐Contreras A , Henriques CM , Selman C , Ferreiro E , Gonos ES , Trejo JL , Misra J , Rasmussen LJ , Xapelli S , Ellam T , Bellantuono I (2016) Interventions for age‐related diseases: shifting the paradigm. Mech. Ageing Dev. 160, 69–92. 27693441 10.1016/j.mad.2016.09.009

[acel12564-bib-0032] Gauldie J (2007) Inflammation and the aging process: devil or angel. Nutr. Rev. 65, S167–S169. 18240542 10.1111/j.1753-4887.2007.tb00356.x

[acel12564-bib-0033] Gruber HE , Hoelscher GL , Ingram JA , Morton DS , Hanley EN Jr (2013) Human annulus cells regulate PAPP‐A and IGFBP‐4 expression, and thereby insulin‐like growth factor bioavailability, in response to proinflammatory cytokine exposure *in vitro* . Connect. Tissue Res. 54, 432–438. 24060054 10.3109/03008207.2013.848200

[acel12564-bib-0034] Hansson GK , Libby P (2006) The immune response in atherosclerosis: a double‐edged sword. Nat. Rev. Immunol. 6, 508–519. 16778830 10.1038/nri1882

[acel12564-bib-0035] Harrington SC , Simari RD , Conover CA (2007) Genetic deletion of pregnancy‐associated plasma protein‐A is associated with resistance to atherosclerotic lesion development in apolipoprotein E‐deficient mice challenged with a high‐fat diet. Circ. Res. 100, 1696–1702. 17510462 10.1161/CIRCRESAHA.106.146183

[acel12564-bib-0036] Hill CM , Arum O , Boparai RK , Wang F , Fang Y , Sun LY , Masternak MM , Bartke A (2015) Female PAPP‐A knockout mice are resistant to metabolic dysfunction induced by high‐fat/high‐sucrose feeding at middle age. Age 37, 9765. 25953669 10.1007/s11357-015-9765-1 PMC4424199

[acel12564-bib-0501] Huang J , Tabata S , Kakiuchi S , Van The T , Goto H , Hanibuchi M , Nishioka Y (2013) Identification of pregnancy‐associated plasma protein A as a migration‐promoting gene in malignant pleural mesothelioma cells: a potential therapeutic target. Oncotarget 4, 1172–1184. 23896451 10.18632/oncotarget.1126 PMC3787149

[acel12564-bib-0037] Jensen MD (2008) Role of body fat distribution and the metabolic complications of obesity. J. Clin. Endocrinol. Metab. 93, S57–S63. 18987271 10.1210/jc.2008-1585 PMC2585758

[acel12564-bib-0038] Juengel JL , Haydon LJ , Mester B , Thomson BP , Beaumont M , Eckery DC (2010) The role of IGFs in the regulation of ovarian follicular growth in the brushtail possum (*Trichosurus vulpecula*). Reproduction 140, 295–303. 20522481 10.1530/REP-10-0142

[acel12564-bib-0039] Junnila RK , List EO , Berryman DE , Murrey JW , Kopchick JJ (2013) The GH/IGF‐1 axis in ageing and longevity. Nat. Rev. Endocrinol. 9, 366–376. 23591370 10.1038/nrendo.2013.67 PMC4074016

[acel12564-bib-0040] Katic M , Kahn CR (2005) The role of insulin and IGF‐1 signaling in longevity. Cell. Mol. Life Sci. 62, 320–343. 15723168 10.1007/s00018-004-4297-y PMC11924452

[acel12564-bib-0041] Kjaer‐Sorensen K , Engholm DH , Kamei H , Morch MG , Kristensen AO , Zhou J , Conover CA , Duan C , Oxvig C (2013) Pregnancy‐associated plasma protein A (PAPP‐A) modulates the early developmental rate in zebrafish independently of its proteolytic activity. J. Biol. Chem. 288, 9982–9992. 23430244 10.1074/jbc.M112.426304 PMC3617297

[acel12564-bib-0042] Laursen LS , Kjaer‐Sorensen K , Andersen MH , Oxvig C (2007) Regulation of insulin‐like growth factor (IGF) bioactivity by sequential proteolytic cleavage of IGF binding protein‐4 and ‐5. Mol. Endocrinol. 21, 1246–1257. 17312271 10.1210/me.2006-0522

[acel12564-bib-0043] Lawrence JB , Oxvig C , Overgaard MT , Sottrup‐Jensen L , Gleich GJ , Hays LG , Yates JR III , Conover CA (1999) The insulin‐like growth factor (IGF)‐dependent IGF binding protein‐4 protease secreted by human fibroblasts is pregnancy‐associated plasma protein‐A. Proc. Natl Acad. Sci. USA 96, 3149–3153. 10077652 10.1073/pnas.96.6.3149 PMC15910

[acel12564-bib-0044] Li M , Li Y , Lu L , Wang X , Gong Q , Duan C (2009) Structural, gene expression, and functional analysis of the fugu (*Takifugu rubripes*) insulin‐like growth factor binding protein‐4 gene. Am. J. Physiol. Regul. Integr. Comp. Physiol. 296, R558–R566. 19091910 10.1152/ajpregu.90439.2008

[acel12564-bib-0045] Mader JR , Resch ZT , McLean GR , Mikkelsen JH , Oxvig C , Marler RJ , Conover CA (2013) Mice deficient in PAPP‐A show resistance to the development of diabetic nephropathy. J. Endocrinol. 219, 51–58. 23881937 10.1530/JOE-13-0167 PMC3820014

[acel12564-bib-0046] Mansfield AS , Visscher DW , Hart SN , Wang C , Goetz MP , Oxvig C , Conover CA (2014) Pregnancy‐associated plasma protein‐A expression in human breast cancer. Growth Horm. IGF Res. 24, 264–267. 25468445 10.1016/j.ghir.2014.10.007 PMC4308469

[acel12564-bib-0047] Marzetti E , Lees HA , Wohlgemuth SE , Leeuwenburgh C (2009) Sarcopenia of aging: underlying cellular mechanisms and protection by calorie restriction. BioFactors 35, 28–35. 19319843 10.1002/biof.5 PMC5992495

[acel12564-bib-0048] Mazerbourg S , Overgaard MT , Oxvig C , Christiansen M , Conover CA , Laurendeau I , Vidaud M , Tosser‐Klopp G , Zapf J , Monget P (2001) Pregnancy‐associated plasma protein‐A (PAPP‐A) in ovine, bovine, porcine, and equine ovarian follicles: involvement in IGF binding protein‐4 proteolytic degradation and mRNA expression during follicular development. Endocrinology 142, 5243–5253. 10.1210/endo.142.12.8517 11713222

[acel12564-bib-0049] Mikkelsen JH , Gyrup C , Kristensen P , Overgaard MT , Poulsen CB , Laursen LS , Oxvig C (2008) Inhibition of the proteolytic activity of pregnancy‐associated plasma protein‐A by targeting substrate exosite binding. J. Biol. Chem. 283, 16772–16780. 18434323 10.1074/jbc.M802429200

[acel12564-bib-0050] Mikkelsen JH , Resch ZT , Kalra B , Savjani G , Kumar A , Conover CA , Oxvig C (2014) Indirect targeting of IGF receptor signaling *in vivo* by substrate‐selective inhibition of PAPP‐A proteolytic activity. Oncotarget 5, 1014–1025. 24572990 10.18632/oncotarget.1629 PMC4011579

[acel12564-bib-0051] Miller BS , Bronk JT , Nishiyama T , Yamagiwa H , Srivastava A , Bolander ME , Conover CA (2007) Pregnancy associated plasma protein‐A is necessary for expeditious fracture healing in mice. J. Endocrinol. 192, 505–513. 17332520 10.1677/JOE-06-0011

[acel12564-bib-0052] Mohan S , Richman C , Guo R , Amaar Y , Donahue LR , Wergedal J , Baylink DJ (2003) Insulin‐like growth factor regulates peak bone mineral density in mice by both growth hormone‐dependent and ‐independent mechanisms. Endocrinology 144, 929–936. 12586770 10.1210/en.2002-220948 PMC2923925

[acel12564-bib-0053] Ning Y , Schuller AGP , Conover CA , Pintar JE (2008) Insulin‐like growth factor (IGF) binding protein‐4 is both a positive and negative regulator of IGF activity *in vivo* . Mol. Endocrinol. 22, 1213–1225. 18258685 10.1210/me.2007-0536 PMC2366183

[acel12564-bib-0054] Oxvig C (2015) The role of PAPP‐A in the IGF system: location, location, location. J. Cell Commun. Signal. 9, 177–187. 25617049 10.1007/s12079-015-0259-9 PMC4458251

[acel12564-bib-0055] Pan H , Hanada S , Zhao J , Mao L , Ma MZ (2012) Protein secretion is required for pregnancy‐associated plasma protein‐A to promote lung cancer growth *in vivo* . PLoS ONE 7, e48799. 23152806 10.1371/journal.pone.0048799 PMC3494721

[acel12564-bib-0056] Perrini S , Leonardini A , Laviola L , Giorgino F (2008) Biological specificity of visceral adipose tissue and therapeutic intervention. Arch. Physiol. Biochem. 114, 277–286. 18946788 10.1080/13813450802334752

[acel12564-bib-0057] Phang D , Rehage M , Bonafede B , Hou D , Xing W , Mohan S , Wergedal JE , Qin X (2010) Inactivation of insulin‐like‐growth factors diminished the anabolic effects of pregnancy‐associated plasma protein‐A (PAPP‐A) on bone in mice. Growth Horm. IGF Res. 20, 192–200. 20144555 10.1016/j.ghir.2010.01.001

[acel12564-bib-0502] Prithviraj P , Anaka M , McKeown SJ , Permazel M , Walkiewicz M , Cebon J , Behren A , Jayachandran A (2015) Pregnancy associated plasma protein‐A links pregnancy and melanoma progression by promoting cellular migration and invasion. Oncotarget 6, 15953–15965. 25940796 10.18632/oncotarget.3643 PMC4599249

[acel12564-bib-0058] Qin X , Sexton C , Byun D , Strong DD , Baylink DJ , Mohan S (2002) Differential regulation of pregnancy‐associated plamsma protein (PAPP)‐A during pregnancy in human and mouse. Growth Horm. IGF Res. 12, 359–366. 12213189 10.1016/s1096-6374(02)00046-1

[acel12564-bib-0059] Qin X , Wergedal JE , Rehage M , Tran K , Newton J , Lam P , Baylink DJ , Mohan S (2006) Pregnancy‐associated plasma protein‐A increases osteoblast proliferation *in vitro* and bone formation *in vivo* . Endocrinology 147, 5653–5661. 16946002 10.1210/en.2006-1055 PMC2904517

[acel12564-bib-0060] Rehage M , Mohan S , Wergedal JE , Bonafede B , Tran K , Hou D , Phang D , Kumar A , Qin X (2007) Transgenic overexpression of pregnancy‐associated plasma protein‐A increases the somatic growth and skeletal muscle mass in mice. Endocrinology 148, 6176–6185. 17901236 10.1210/en.2007-0274

[acel12564-bib-0061] Resch ZT , Chen BK , Bale LK , Oxvig C , Overgaard MT , Conover CA (2004) Pregnancy‐associated plasma protein a gene expression as a target of inflammatory cytokines. Endocrinology 145, 1124–1129. 14657012 10.1210/en.2003-1313

[acel12564-bib-0062] Salim H , Arvanitis A , de Petris L , Kanter L , Haag P , Zovko A , Ozata DM , Lui W‐O , Lundholm L , Zhivotovsky B , Lewensohn R , Viktorsson K (2013) miRNA‐214 is related to invasiveness of human non‐small cell lung cancer and directly regulates alpha protein kinase 2 expression. Genes Chromosom. Cancer 52, 895–911. 23929716 10.1002/gcc.22085

[acel12564-bib-0063] Smith GCS , Stenhouse EJ , Crossley JA , Aitken DA , Cameron AD , Connor JM (2002) Early‐pregnancy origins of low birth weight. Nature 417, 916. 12087395 10.1038/417916a

[acel12564-bib-0064] Soe R , Overgaard MT , Thomsen AR , Laursen LS , Olsen IM , Sottrup‐Jensen L , Haaning J , Giudice LC , Conover CA , Oxvig C (2002) Expression of recombinant murine pregnancy‐associated plasma protein‐A (PAPP‐A) and a novel variant (PAPP‐Ai) with differential proteolytic activity. Eur. J. Biochem. 269, 2247–2256. 11985604 10.1046/j.1432-1033.2002.02883.x

[acel12564-bib-0065] Sohn HY , Krotz F (2006) Cyclooxygenase inhibition and atherothrombosis. Curr. Drug Targets 7, 1275–1284. 17073588 10.2174/138945006778559102

[acel12564-bib-0066] Swindell WR , Masternak MM , Bartke A (2010) *In vivo* analysis of gene expression in long‐lived mice lacking the pregnancy‐associated plasma protein A (PappA) gene. Exp. Gerontol. 45, 366–374. 20197085 10.1016/j.exger.2010.02.009 PMC2860881

[acel12564-bib-0067] Tang S‐L , Chen W‐J , Yin K , Zhao G‐J , Mo Z‐C , Lu Y‐C , Ouyang X‐P , Yu X‐H , Kuang H‐J , Jiang Z‐S , Fu Y‐C , Tang C‐K (2012) PAPP‐A negatively regulates ABCA1, ABCG1 and SR‐B1 expression by inhibiting LXRα through the IGF‐I‐mediated signaling pathway. Atherosclerosis 222, 344–354. 22503545 10.1016/j.atherosclerosis.2012.03.005

[acel12564-bib-0068] Tchoukalova YD , Nathanielsz PW , Conover CA , Smith SR , Ravussin E (2009) Regional variation in adipogenesis and IGF regulatory proteins in the fetal baboon. Biochem. Biophys. Res. Comm. 380, 679–683. 19285021 10.1016/j.bbrc.2009.01.149 PMC2733229

[acel12564-bib-0069] Thomsen J , Hjortebjerg R , Espelund U , Ortoft G , Vestergaard P , Magnusson NE , Conover CA , Tramm T , Hager H , Hogdall C , Hogdall E , Oxvig C , Frystyk J (2015) PAPP‐A proteolytic activity enhances IGF bioactivity in ascites from women with ovarian carcinoma. Oncotarget 6, 32266–32278. 26336825 10.18632/oncotarget.5010 PMC4741676

[acel12564-bib-0070] Vallejo AN , Michel JJ , Bale LK , Lemster BH , Borghesi L , Conover CA (2009) Resistance to age‐dependent thymic atrophy in long‐lived mice that are deficient in pregnancy‐associated plasma protein A. Proc. Natl Acad. Sci. USA 106, 11252–11257. 19549878 10.1073/pnas.0807025106 PMC2700140

[acel12564-bib-0071] Wajchenberg BL (2000) Subcutaneous and visceral adipose tissue: their relation to the metabolic syndrome. Endocr. Rev. 21, 697–738. 11133069 10.1210/edrv.21.6.0415

